# Indeterminate dental pain: clinical characteristics and neurovascular compression; a retrospective case comparative study

**DOI:** 10.3389/fpain.2025.1694598

**Published:** 2025-11-17

**Authors:** Kiyokazu Iwawaki, Motoko Watanabe, Yasuyuki Kimura, Chizuko Maeda, Chihiro Takao, Risa Tominaga, Takayuki Suga, Trang Thi Huyen Tu, Takahiko Nagamine, Akira Toyofuku

**Affiliations:** 1 Department of Psychosomatic Dentistry, Graduate School of Medical and Dental Sciences, Institute of Science Tokyo, Tokyo, Japan; 2 Division of Special Needs Dentistry and Orofacial Pain and Clinical Science, Tokyo Dental College, Tokyo, Japan; 3 Department of Basic Dental Science, Faculty of Odonto-Stomatology, University of Medicine and Pharmacy at Ho Chi Minh City, Ho Chi Minh City, Vietnam; 4 Department of Psychiatric Internal Medicine, Sunlight Brain Research Center, Yamaguchi, Japan

**Keywords:** persistent idiopathic dentoalveolar pain, trigeminal neuralgia, neurovascular compression, atypical odontalgia, persistent idiopathic facial pain, central sensitization, pain catastrophizing, somatic symptom

## Abstract

**Objective:**

Non-odontogenic toothache, which is characterized by tooth pain without corresponding dental abnormality, is occasionally indeterminate due to its complicated persistent teeth, dentoalveolar and/or facial pain, specifically between patients with persistent idiopathic dentoalveolar pain (PIDAP) and those with trigeminal neuralgia (TN), accompanied by atypical sensations. This study aimed to clarify clinical characteristics in this patient population and to identify clinical real-world factors for differentiation.

**Methods:**

All clinical data were retrospectively collected. Totally 340 patients, who were referred to our department with undiagnosed complicated persistent pain, were involved in the comparative analysis, depending on symptoms’ laterality, and 149 patients with unilateral symptoms were involved, depending on the presence of neurovascular compression (NVC) of trigeminal nerves and final diagnosis of PIDAP or TN.

**Results:**

Patients with bilateral symptoms (*n* = 105) presented more severe affected pain sensations with higher pain catastrophizing compared to patients with unilateral symptoms (*n* = 234, *p* = 0.022). NVC was observed in 84 patients (56.4%); however, no significant difference in clinical features was observed depending on the presence of NVC. While patients with TN (*n* = 26) presented significantly stronger “shooting” and “stabbing” pain (*p* = 0.004, *p* = 0.006, respectively) with more severe NVC condition (*p* = 0.033), patients with PIDAP (*n* = 123) showed significantly higher scores in the central sensitization inventory (*p* < 0.001) and somatic symptom scales-8 (*p* = 0.004).

**Conclusion:**

These results suggest that relying solely on examining the presence of NVC is insufficient to distinguish PIDAP and TN in this patient population, but careful assessment of pain quality, pain catastrophizing, central sensitization, and somatic symptoms, besides detailed neurovascular conditions, is crucial.

## 1 Introduction

Non-odontogenic toothache is characterized by tooth pain without dental abnormalities and a lack of improvement with conventional dental procedures (1, 2). Tooth extractions are sometimes repeated due to a persistent toothache, but this does not alleviate the pain. Pain sensations in non-odontogenic toothache are distributed not only to the teeth and dentoalveolar area but also to the face, with varying symptom severity. Multiple factors, including ephapse, peripheral/central sensitization, and alternation of neurotransmitters in the central nervous system, have been considered to complicate interactions and to lead to a wide range of symptoms' characteristics and severity. Non-odontogenic toothache, specifically, persistent idiopathic dentoalveolar pain (PIDAP) and trigeminal neuralgia (TN), sometimes present similar unilateral persistent pain, which makes them extremely difficult to distinguish.

PIDAP is pain in the teeth or alveolar region without any clinical or radiographic abnormality, and its pain may radiate to the face on some occasions, according to the International Classification of Orofacial Pain (ICOP) (3, 4). The typical descriptions of PIDAP pain are “dull, throbbing pain” or “heavy, aching pain.” (5, 6). According to the International Classification of Headache Disorders, 3rd Edition, it is defined as “the term *atypical odontalgia* has been applied to a continuous pain in one or more teeth or in a tooth socket after extraction, in the absence of any usual dental cause”, and classified as a subtype of persistent idiopathic facial pain (PIFP) (7).

TN is typically characterized by paroxysmal, intermittent, electric shock-like pain localized within the trigeminal nerve distribution. In ICOP, TN is classified into three categories: classic trigeminal neuralgia caused by neurovascular compression (NVC) at the trigeminal nerve root entry zone (REZ), secondary TN caused by other diseases, and idiopathic TN in which no obvious cause, such as NVC at the trigeminal REZ, is identified (8–10). Both classic and idiopathic TN can be further subdivided depending on with/without continuous pain (7, 10).

Moreover, besides continuous pain, some patients with TN exhibit atypical pain symptoms, including dull aching, burning sensations (10, 11), and a persistent toothache (11–13). Such atypical sensations of TN can be confusingly similar to PIDAP. Because both TN and PIDAP are non-odontogenic toothaches without corresponding dental abnormality, and present persistent pain in orofacial regions, including teeth, their distinction would be difficult. Consequently, establishing a precise diagnosis and treatment is challenging and important in this patient population.

NVC of trigeminal nerves is one of the causes of trigeminal neuralgia, reporting that 85% of trigeminal neuralgia patients presented NVC (14). However, the previous study reported that approximately 40% of patients with PIDAP also demonstrate NVC (15). It is unclear if the presence or absence of NVC could differentiate TN with atypical sensations from PIDAP in this patient population. Pharmacologically, the efficacy of carbamazepine for trigeminal neuralgia has been well known (8, 16), while tricyclic antidepressants, such as amitriptyline, are generally used as the first-line medication for PIDAP (5, 17). Therefore, determining a correct diagnosis is crucial for establishing a more accurate treatment strategy.

Patients in our department experience pain and unpleasant sensations in their orofacial regions, including the teeth, dentoalveolar, and facial areas, without any identifiable dental abnormalities explaining their complaints. These patients are often referred from dental or oral surgery clinics and internal medicine or psychiatry departments after clinical examinations fail to find any corresponding abnormality. The primary aim of this study was to clarify the clinical features, including pain characteristics and the presence of NVC on magnetic resonance imaging (MRI), in this patient population, and the secondary aim was to identify clinical factors that differentiate PIDAP from TN with atypical sensations.

## 2 Methods

### 2.1 Subjects

**Figure 1:**
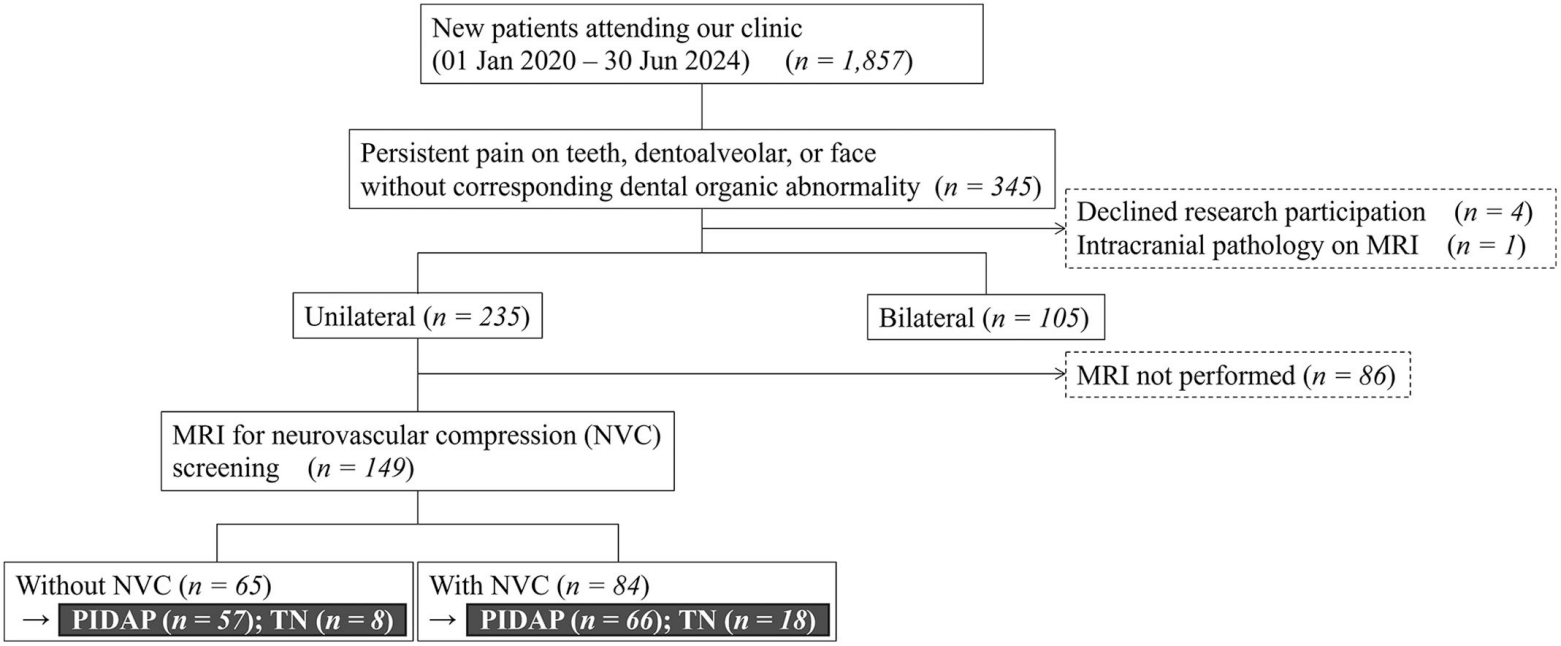
Flowchart for selection of patients. Among 1,857 patients who were referred to our department with undiagnosed complicated teeth, dentoalveolar, and/or facial pain, 340 patients were involved in the analysis, depending on the symptoms’ laterality, and 149 of them were involved depending on the presence of neurovascular compression. Further analysis was conducted depending on the final diagnosis. MRI, magnetic resonance imaging; NVC, neurovascular compression; PIDAP, persistent idiopathic dentoalveolar pain; TN, trigeminal neuralgia.

Among 1,857 new outpatients at the Department of Psychosomatic Dentistry at Institute of Science Tokyo Hospital, between January 2020 and June 2024, 345 individuals who complained of teeth, dentoalveolar and/or facial pain without any corresponding dental organic abnormality and who did not improve with conventional dental procedures at primary/secondary medical institutions were included (Figure 1). The exclusion criteria were patients who did not consent to participate in this study (*n* = 4) and who exhibited organic brain diseases on MRI (*n* = 1). Consequently, 340 patients underwent the first analysis to examine the clinical difference depending on symptoms laterality: unilateral or bilateral.

Further, 149 patients, who had unilateral symptoms and underwent MRI to assess the NVC at REZ, were involved in the secondary analysis. While patients with NVC were categorized into the “with NVC” group, the others without NVC were categorized into the “without NVC” group to analyze between-group clinical differences. Moreover, the analysis of the clinical features relating to diagnosis and responses to pharmacotherapy with over six months of follow-up was conducted depending on the final diagnosis. The final diagnosis was made by at least one board-certified psychosomatic dentist from the Japanese Society of Psychosomatic Dentistry, in accordance with ICOP (3).

This study was conducted following the principles of the Helsinki Declaration of the World Medical Association, and approved by the Ethics Committee of the Institute of Science Tokyo, Faculty of Dentistry (Approval No. D2022-056). Written informed consent was obtained from all patients.

### 2.2 Data analysis

Based on outpatient medical records, retrospective data collection and analysis were conducted in the following data: age, sex, duration of illness, comorbid psychiatric disorders, number of remaining teeth excluding wisdom teeth, the scores of clinical questionnaires at the initial visit, regions of pain, presence or absence and degree of NVC, response to medications, surgical outcomes, presence or absence of dental treatment that triggered the onset, and use of benzodiazepine medications at the initial consultation. Psychiatric comorbidities, if any, were recorded according to the referral letter from the attending psychiatrist based on the Diagnostic and Statistical Manual of Mental Disorders, Fifth Edition (DSM-5) by the American Psychiatric Association (18).

For clinical questionnaires, visual analogue scale (VAS), the short-form McGill pain questionnaire (SF-MPQ), central sensitization inventory (CSI), somatic symptom scale-8 (SSS-8), Zung's self-rating depression scale (SDS), and pain catastrophizing scale (PCS) were used. Pain intensity was evaluated using VAS. Patients were asked to mark their current pain intensity on a 100 mm line (0: the absence of pain, 100: the strongest pain ever experienced), and the measured value was recorded. Pain quality was evaluated using SF-MPQ (19, 20), which comprises 11 sensory descriptors (throbbing, shooting, stabbing, sharp, cramping, gnawing, burning, aching, heavy, tender, splitting) and 4 affective descriptors (tiring-exhausting, sickening, fearful, punishing-cruel). Patients rate each of these 15 descriptors on a 4-point scale (none, mild, moderate, severe). The central sensitization, which involves a heightened pain sensitivity, was assessed using CSI (21). CSI consists of PART A, with 25 items rated on a 5-point Likert scale, and PART B inquires about past diagnoses of 10 diseases. In this study, only PART A was used in the analysis. SSS-8 (22), which also relates to central sensitization syndrome (23), was used to evaluate the severity of somatic symptoms. Depression at the initial visit was evaluated using SDS (24), and catastrophic thinking in pain, which relates to pain chronicity, was assessed using PCS (25).

### 2.3 MRI acquisition and assessment of NVC

**Figure 2:**
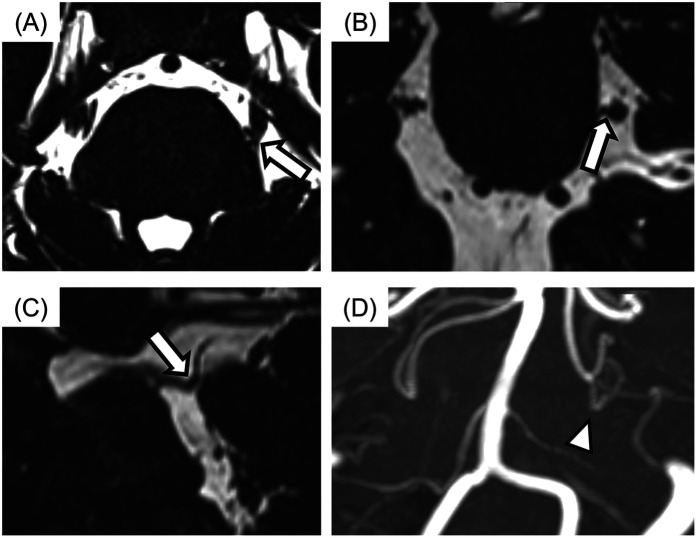
The images of neurovascular compression (NVC) with the trigeminal nerve. **(A)** Axial view, **(B)** sagittal view, **(C)** coronal view, **(D)** display of 3D time-of-flight magnetic resonance angiography (3D-TOF MRA). The case with no cerebrospinal fluid between the trigeminal nerve and blood vessel was assessed for NVC presence (arrows). Responsible blood vessel: the superior cerebellar artery (arrowhead) was revealed by using 3D-TOF MRA.

**Figure 3:**
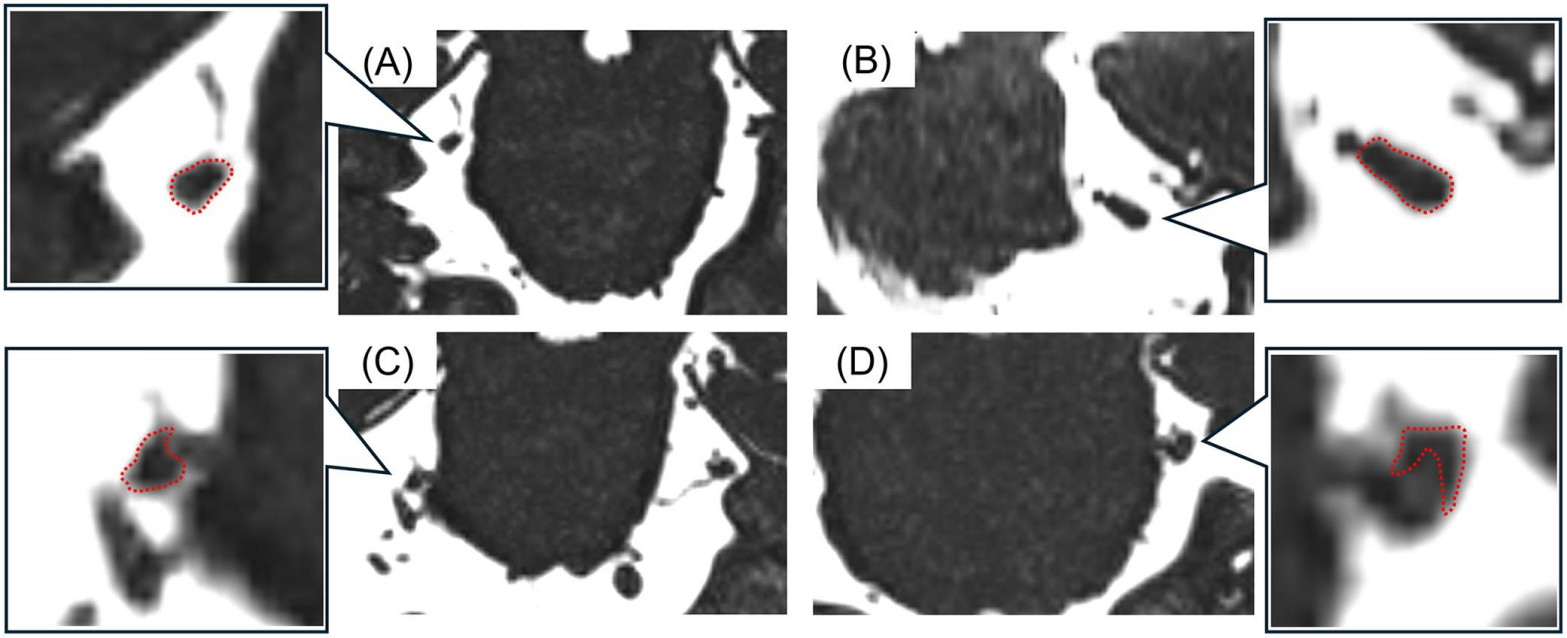
The images of detailed neurovascular compression (NVC). **(A)** Without NVC, **(B)** contact, **(C)** compression, **(D)** displacement. The detailed NVC conditions were assessed according to the shapes and courses of the trigeminal nerves (surrounded with a red dotted line).

All MR images were obtained at the REZ of the trigeminal nerve by using a three-tesla MRI scanner (Magnetom Spectra, Siemens, Germany) with a 16-channel head coil according to our previous study (15, 26). MR angiography (MRA) was obtained using 3D time-of-flight (3D-TOF) MRA with the following parameters: repetition time/echo time, flip angle = 24/3.9 ms, 18°; field of view 160 mm × 160 mm; matrix 320 × 192; section thickness 0.5 mm; and slab number 3. MR cisternography was obtained using 3D-constructive interference in steady-state (3D-CISS) MRA with the following parameters: repetition time/echo time, flip angle = 7.4/3.7 ms, 50°; field of view 160 mm × 160 mm; matrix 320 × 320. These MR images were reconstructed to a voxel size of 0.5 mm × 0.5 mm × 0.5 mm and slab thickness of 44 mm. All 3D-TOF and 3D-CISS images were displayed in triplanar views (transverse, coronal, and sagittal views) on the visualization system. Images were assessed for NVC presence by two experienced radiology specialists blinded to the laterality of symptoms. NVC presence was defined as contact between the blood vessel and the trigeminal nerve at REZ. When cerebrospinal fluid was not present between them in the 3D-CISS, it was defined as “with NVC” (Figure 2). In case of disagreements or uncertainties, whether there was contact or not, it is regarded as “without NVC”. Regarding details of NVC, the blood vessels involved in NVC and the degree of NVC, whether with only simple contact or with compression or displacement, were assessed (Figure 3).

### 2.4 Treatment response

In patients who were finally diagnosed with PIDAP or TN and who underwent pharmacotherapy with amitriptyline or carbamazepine, medication responses were evaluated. The global improvement section of the Clinical Global Impression (CGI) (27) was used to assess prognosis. Following previous studies (17, 28), the time point when patients achieved “marked improvement” or “moderate improvement” was defined as “clinical improvement,” and the duration (days) until the improvement and the medication dose at “clinical improvement” were recorded. The interaction between the medication dose at “clinical improvement” and the initial VAS scores was also analyzed. For patients with TN who underwent microvascular decompression surgery, postoperative status was evaluated retrospectively based on medical records.

### 2.5 Statistical analysis

All data were analyzed using Student's *t*-test, the Mann–Whitney *U*-test, or the Chi-square test for between-group comparisons. Specifically, age, duration of illness, number of remaining teeth, initial VAS, and mean scores on various questionnaires (CSI, SDS, PCS, and SSS-8) were analyzed using Student's *t*-test with Bonferroni correction. Distributions of each item in SF-MPQ, CSI, and SSS-8 were analyzed using the Mann–Whitney *U* test with Bonferroni correction, and the frequencies of psychiatric comorbidity, headache history, pain location, presence or absence of NVC, details of NVC, triggering dental treatment, and benzodiazepine use at the initial visit were analyzed using the Chi-square test. For the between-group analysis, depending on the final diagnosis, ANCOVA controlled by age was conducted in comparisons of duration of illness, the number of teeth, and the scores of VAS, CSI, SDS, PCS, and SSS-8. Correlations between medication dose at the time of improvement and initial VAS were examined using Pearson's correlation analysis. Analyses were performed using IBM SPSS Statistics Ver.26 (IBM Corp., New York, USA). All data are presented as mean ± standard deviation, median [first quartile, third quartile], or number (%). A *p*-value < 0.05 was considered statistically significant.

## 3 Results

### 3.1 Comparison of unilateral vs. bilateral symptoms

**Figure 4:**
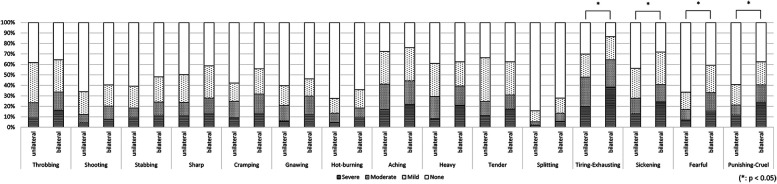
The differences in pain characteristics of the short-form mcGill pain questionnaire between patients with bilateral and unilateral pain. Patients with bilateral pain symptoms had significantly higher scores in “tiring-exhausting” (*p* = 0.001), “sickening” (*p* = 0.030), “fearful” (*p* < 0.001), and “punishing-cruel” (*p* < 0.001) compared to patients with unilateral symptoms.

In the first analysis comparing patients with unilateral symptoms (*n* = 235) and patients with bilateral symptoms (*n* = 105), the PCS score was significantly higher in the bilateral group (31.7 ± 12.1, 34.9 ± 10.9, *p* = 0.022, respectively). No significant between-group difference was found in other clinical characteristics (Table 1); however, the evaluation of detailed pain quality using SF-MPQ showed significant differences in the distributions of “tiring-exhausting” (*p* = 0.001), “sickening” (*p* = 0.030), “fearful” (*p* < 0.001), and “punishing-cruel” (*p* < 0.001), all of which were more frequently recognized in patients with bilateral group (Figure 4).

**Table 1:** Comparison according to symptom laterality.

Clinical characteristics	Unilateral (*n* = 235)	Bilateral (*n* = 105)	*p*-values
Female (%)^§^	199 (84.7)	88 (83.8)	0.872
Age (years old)^†^	55.8 ± 15.1	53.2 ± 16.7	0.168
Duration of illness (month)^†^	41.7 ± 53.4	55.2 ± 80.2	0.115
The number of teeth^†^	24.5 ± 5.5	25.1 ± 5.3	0.299
VAS^†^	54.8 ± 28.0	59.3 ± 28.0	0.179
Psychological questionnaires			
CSI^†^	32.9 ± 21.1	36.8 ± 20.7	0.112
SDS^†^	45.2 ± 10.6	46.7 ± 11.4	0.245
PCS^†^	31.7 ± 12.1	34.9 ± 10.9	**0** **.** **022**
SSS-8^†^	9.8 ± 6.1	10.3 ± 6.5	0.424
Psychiatric comorbidities (%)^§^	94 (40.0)	52 (49.5)	0.123
Headache history (%)^§^	130 (55.3)	55 (52.4)	1.000
Triggered by dental procedures (%)^§^	126 (53.6)	52 (49.5)	0.557

VAS, visual analogue scale; CSI, central sensitization inventory; SDS, the Zung's self-rating depression scale; PCS, pain catastrophizing scale; SSS-8, somatic symptom scale-8.

The data are presented as mean ± standard deviation (SD) or number (%).

Bold numbers indicate *p*-values <0.05.

§ Chi-square test.

† Student *t*-test.

### 3.2 Comparison according to the presence or absence of NVC

**Figure 5:**
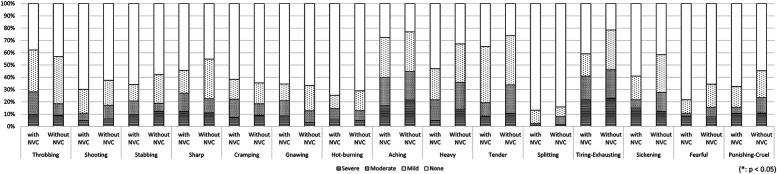
The differences in pain characteristics in the short-form mcGill pain questionnaire between patients with and without neurovascular compression (NVC). There was no significant difference in pain descriptors depending on the presence of NVC. NVC, neurovascular compression.

Among 149 patients who experienced unilateral symptoms and underwent MRI to evaluate NVC at REZ, NVC was observed in 84 patients (56.4%). No significant differences were observed in the pain distributions and other clinical features regardless of the presence of NVC (Figure 5, Table 2).

**Table 2:** Comparison according to the presence or absence of NVC.

Clinical characteristics		With NVC (*n* = 84)	Without NVC (*n* = 65)	*p*-values
Female (%)^§^		72 (85.7)	57 (87.6)	0.811
Age (years old)^†^		57.7 ± 15.0	56.1 ± 14.2	0.521
Duration of illness (month)^†^		39.4 ± 61.0	39.3 ± 47.0	0.989
The number of teeth^†^		24.2 ± 6.3	24.6 ± 4.8	0.670
VAS^†^		50.3 ± 28.6	58.0 ± 26.3	0.095
Distribution of pain location (%, multiple answers included)				
Maxillary right (%)^§^	Molars	15 (17.9)	18 (27.7)	0.168
	Anterior teeth	4 (4.8)	1 (1.5)	0.387
Maxillary left (%)^§^	Molars	20 (23.8)	19 (29.2)	0.460
	Anterior teeth	5 (6.0)	7 (10.8)	0.366
Mandibular right (%)^§^	Molars	17 (20.2)	10 (15.4)	0.402
	Anterior teeth	0 (0.0)	3 (4.6)	0.081
Mandibular left (%)^§^	Molars	21 (25.0)	10 (15.3)	0.162
	Anterior teeth	1 (1.2)	1 (1.2)	1.000
Facial pain (%)^§^		14 (16.7)	12 (19.3)	0.830
Psychological questionnaires (mean ± SD)				
CSI^†^		29.1 ± 15.4	29.2 ± 13.7	0.957
SDS^†^		44.8 ± 8.3	44.6 ± 10.0	0.894
PCS^†^		30.0 ± 11.1	31.9 ± 11.1	0.302
SSS-8^†^		8.8 ± 5.9	9.7 ± 5.4	0.306
Psychiatric comobidities (%)^§^		28 (33.3)	23 (35.3)	0.862
Depressive disorders		10	8	
Anxiety disorders		7	3	
Somatic symptom and related disorder		3	4	
Insomnia disorder		3	4	
Adjustment disorders		2	2	
Obsessive-Compulsive and Related disorders		1	0	
Bipolar and related disorders		2	2	
Neurocognitive disorders		2	0	
Schizophrenia		1	1	
Eating disorder		0	1	
Borderline Personally Disorder		1	0	
Diagnosis is unspecified		1	1	
Headache history (%)^§^		49 (58.3)	33 (50.8)	0.408
Triggered by dental procedures (%)^§^		39 (46.4)	29 (45.3)	0.868

NVC, neurovascular compression; VAS, visual analogue scale; CSI, central sensitization inventory; SDS, the Zung's self-rating depression scale; PCS, pain catastrophizing scale; SSS-8, somatic symptom scale-8.

The data are presented as mean ± standard deviation (SD) or number (%).

Bold numbers indicate *p*-values <0.05.

§ Chi-square test.

† Student *t*-test.

### 3.3 Comparison according to final diagnosis

#### 3.3.1 Demographic characteristics

Based on the final diagnosis, 123 patients were classified as PIDAP and 26 patients as TN (Table 3). NVC was observed in 53.7% of patients with PIDAP and 69.2% of patients with TN without a significant between-group difference (*p* = 0.192). However, compressed or displaced trigeminal nerves were more significantly detected in patients with TN compared to those with PIDAP (*p* = 0.033), while no significant between-group difference was detected in the responsible blood vessels or NVC laterality (*p* = 0.402, *p* = 0.807, respectively). The mean age of patients with TN was significantly higher than that of patients with PIDAP (PIDAP: 55.2 ± 14.0, TN: 66.0 ± 14.7, *p* < 0.001). Both patient groups showed female predominance with no significant difference in the female ratio (PIDAP: 88.6%, TN: 76.9%, *p* = 0.121). Patients with TN experienced facial pain with significantly higher frequency (PIDAP: 13.0%, TN: 38.5%, *p* = 0.004), and 69.2% of them also reported tooth pain.

**Table 3:** Comparison according to final diagnosis.

Clinical characteristics		PIDAP (*n* = 123)	TN (*n* = 26)	*p*-values
The presence of NVC (%)^§^		66 (53.7)	18 (69.2)	0.192
Degree of NVC (%)^§^				**0.033**
	None	57 (46.3)	8 (30.8)	
	Contact	43 (35.0)	7 (26.9)	
	Compression	20 (16.3)	10 (38.5)	
	Displacement	3 (2.4)	1 (3.8)	
Responsible blood vessels^§^				0.402
	Artery	40	14	
	Vein	19	3	
	Both	7	1	
NVC Laterality^§^				0.807
	Ipsilateral	48	14	
	Contolateral	7	1	
	Bilateral	11	3	
Female (%)^§^		109 (88.6)	20 (76.9)	0.121
Age (years old)^†^		55.2 ± 14.0	66.0 ± 14.7	**<0.001**
Duration of illness (month)^‡^		41.3 ± 51.9	30.0 ± 69.0	0.293
The number of teeth^‡^		25.0 ± 4.8	21.4 ± 8.4	0.117
VAS^‡^		52.5 ± 27.1	59.3 ± 30.8	0.291
Distribution of pain location (%, multiple answers included)				
Tooth and dentoalveolar pain (%)^§^		118 (95.9)	18 (69.2)	**<0.001**
Maxillary right (%)^§^	Molars	29 (23.6)	4 (15.3)	0.444
	Anterior teeth	4 (3.2)	1 (3.8)	0.587
Maxillary left (%)^§^	Molars	35 (28.4)	4 (15.3)	0.222
	Anterior teeth	11 (8.9)	1 (3.8)	0.693
Mandibular right (%)^§^	Molars	22 (17.9)	6 (23.1)	0.582
	Anterior teeth	2 (1.6)	1 (3.8)	0.440
Mandibular left (%)^§^	Molars	28 (22.8)	3 (11.5)	0.289
	Anterior teeth	2 (1.6)	0 (0.0)	1.000
Facial pain (%)		16 (13.0)	10 (38.5)	**0.004**
Psychological questionnaires (mean ± SD)				
CSI^‡^		31.2 ± 14.3	19.4 ± 13.8	**<0.001**
SDS^‡^		45.1 ± 9.0	42.8 ± 9.6	0.222
PCS^‡^		30.7 ± 10.5	31.7 ± 13.5	0.602
SSS-8^‡^		9.9 ± 5.6	6.7 ± 5.8	**0.004**
Psychiatric comobidities (%)^§^		47 (38.2)	4 (15.4)	**0.039**
Depressive disorders		18	0	
Anxiety disorders		9	1	
Somatic symptom and related disorder		6	2	
Insomnia disorder		7	0	
Adjustment disorders		4	0	
Obsessive-Compulsive and Related disorders		1	0	
Bipolar and related disorders		4	0	
Neurocognitive disorders		2	0	
Schizophrenia		2	0	
Eating disorder		1	0	
Borderline Personally Disorder		1	0	
Diagnosis is unspecified		1	1	
Headache history (%)^§^		73 (59.3)	9 (34.6)	**0.029**
Triggered by dental procedures (%)^§^		57 (46.3)	7 (26.9)	0.083
Internal use of benzodiazepines^§^		35 (28.5)	4 (15.4)	0.222
Treatment responses				
Amitriptyline	Improved (%)	54/80 (67.5)	3/7 (42.9)	
	Effective dose	30 [20, 30]	—	
	The duration of taking medication until improvement	44 [35.25, 79]	—	
Carbamazepine	Improved (%)	3/11 (27.3)	11/14 (78.5)	
	Effective dose	—	100 [100, 150]	
	The duration of taking medication until improvement	—	16.0 [8.5, 25.5]	
Micro-vascular decompression		0	4/4 (100)	

PIDAP, persistent idiopathic dentoalveolar pain; TN, trigeminal neuralgia; NVC, neurovascular compression; VAS, visual analogue scale; CSI, central sensitization inventory; SDS, the Zung's self-rating depression scale; PCS, pain catastrophizing scale; SSS-8, somatic symptom scale-8.

The data are presented as mean ± standard deviation (SD), number (%), or median [interquartile range (IQR)].

Bold numbers indicate *p*-values <0.05.

§ Chi-square test.

† Student *t*-test.

‡ Multiple regression analysis controlled with age.

Significantly more patients with PIDAP had psychiatric comorbidities and a history of headache (psychiatric comorbidities: PIDAP: 38.2%, TN: 15.4%, *p* = 0.039; headache history: PIDAP: 59.3%, TN: 34.6%, *p* = 0.029). The most observed psychiatric comorbidities in patients with PIDAP were depressive disorders, and those in patients with TN were somatic symptoms and related disorders. There was no significant difference between groups in the ratio of patients taking benzodiazepines at the initial visit. More patients with PIDAP had dental procedures as an onset trigger (46.3%) compared to patients with PIDAP (26.9%), but there was no significant between-group difference.

#### 3.3.2 Pain quality

**Figure 6:**
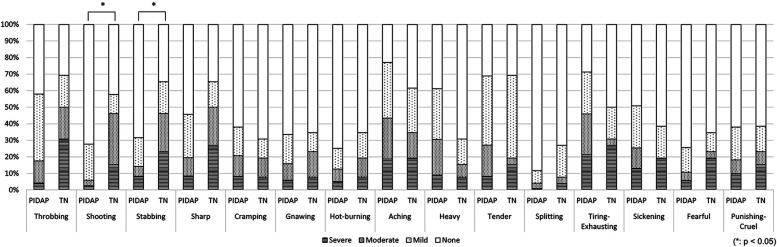
The differences in pain characteristics of the short-form mcGill pain questionnaire between patients with PIDAP and TN. Patients with TN showed significantly more severe “shooting” (*p* = 0.004) and “stabbing” (*p* = 0.006) pain compared to patients with PIDAP. PIDAP, persistent idiopathic dentoalveolar pain; TN, trigeminal neuralgia.

Regarding pain quality evaluated by SF-MPQ, “shooting” (*p* = 0.004) and “stabbing” (*p* = 0.006) were reported significantly more frequently by patients with TN (Figure 6).

#### 3.3.3 Questionnaires

Multiple regression analysis controlled with age revealed that patients with PIDAP showed significantly higher scores in CSI (PIDAP: 31.2 ± 14.3; TN: 19.4 ± 13.8; *p* < 0.001) and SSS-8 (PIDAP: 9.9 ± 5.6; TN: 6.7 ± 5.8; *p* = 0.004) compared to patients with TN, while no significant between-group differences were observed in scores of SDS or PCS (Table 3).

**Figure 7:**
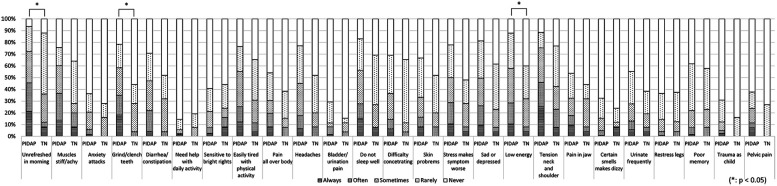
The differences in each item of central sensitization inventory between patients with PIDAP and TN. Patients with PIDAP reported a significantly higher frequency of “unrefreshed in morning” (*p* = 0.017) and “grind/clench teeth” (*p* = 0.005), and “low energy” (*p* = 0.04), compared to patients with TN. PIDAP, persistent idiopathic dentoalveolar pain; TN, trigeminal neuralgia.

**Figure 8:**
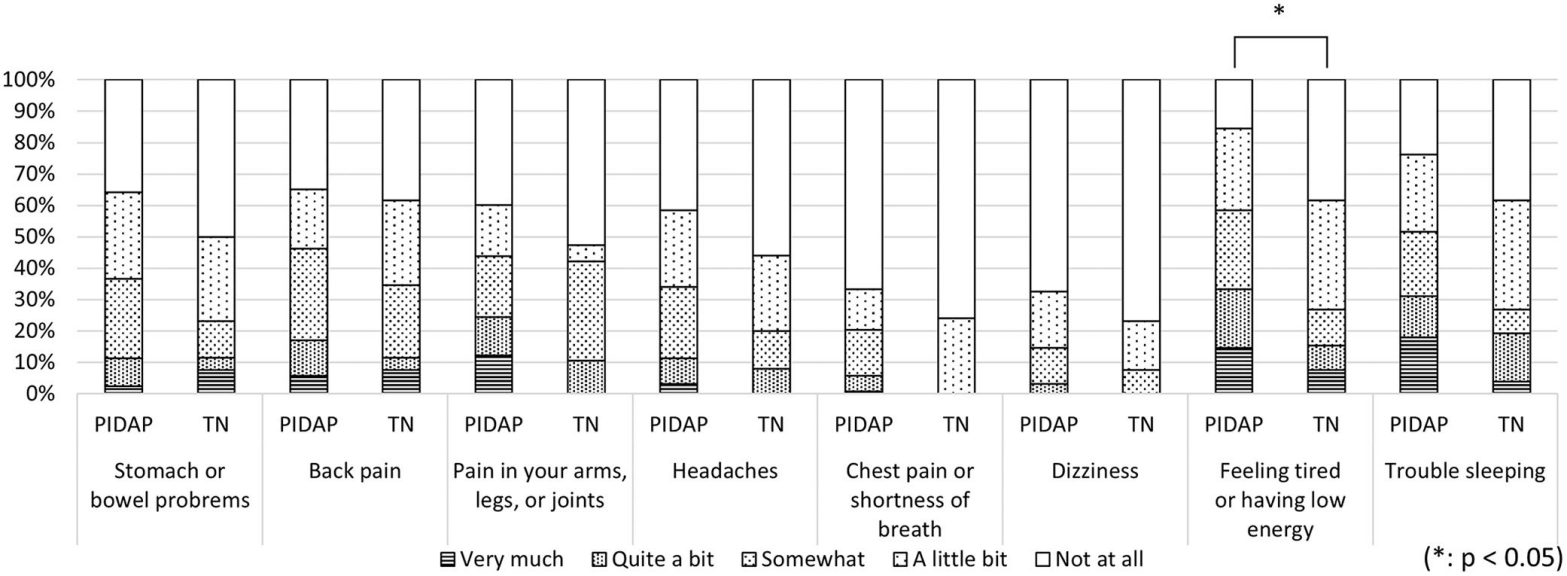
Difference in pain characteristics in somatic symptoms scale-8 between patients with PIDAP and TN. Patients with PIDAP reported a significantly higher frequency of “feeling tired or having low energy” (*p* = 0.024) compared to patients with TN. PIDAP, persistent idiopathic dentoalveolar pain; TN, trigeminal neuralgia.

A more detailed analysis of each CSI item revealed that PIDAP patients reported a significantly higher frequency of the following: “unrefreshed in morning” (*p* = 0.017) and “grind/clench teeth” (*p* = 0.005), and “low energy” (*p* = 0.04), as shown in Figure 7. Similarly, for the SSS-8, PIDAP patients reported a significantly higher frequency of “feeling tired or having low energy” (*p* = 0.024) compared to TN patients (Figure 8).

#### 3.3.4 Treatment response

Among 80 patients with PIDAP who were prescribed amitriptyline, 67.5% (54/80) of patients improved (Table 3). The median duration until improvement was 44 [35.25, 79] days, and the dose at the time of improvement was 30 [20, 30] mg. The maximum dose was 60 mg, and the minimum dose was 5 mg; the longest time to improvement was 182 days, and the shortest was 13 days.

In 14 patients with TN who were prescribed carbamazepine, 78.5% (11/14) showed improvement. The median time to improvement and the dose at that time were 16.0 [8.5, 25.5] days and 100 [100, 150] mg, respectively. The maximum dose administered was 200 mg, the minimum was 50 mg, the longest time to improvement was 61 days, and the shortest was one day (Table 3).

No significant correlation was found between the dose at the time of improvement and the initial VAS score in either group. Meanwhile, carbamazepine was effective in 27.3% (3/11) of PIDAP patients, and amitriptyline was effective in 42.9% (3/7) of TN patients. Of the TN patients with NVC, four underwent microvascular decompression (MVD), and all experienced improvement.

## 4 Discussion

This study focused on patients experienced complicated persistent teeth, dentoalveolar or facial pain without identifiable dental origin abnormality, and revealed that 1) patients with bilateral symptoms present more severe affected pain sensations with higher pain catastrophizing compared to patients with unilateral symptoms, 2) among patients with unilateral symptoms, no significant difference in clinical features was observed depending on the presence of NVC, 3) while patients with TN present significantly stronger “shooting” and “stabbing” pain in SF-MPQ besides more severe condition of NVC, patients with PIDAP showed higher scores in CSI and SSS-8 as well as some items of them. These results suggest that relying solely on examining the presence of NVC is insufficient to distinguish PIDAP and TN in this patient population, but careful assessment of pain catastrophizing, central sensitization, and somatic symptoms, as well as pain quality, is crucial.

The widespread pain, as bilateral involvement, may amplify catastrophic thinking, contributing to a heightened affective experience of pain. In this study, patients with bilateral pain had significantly higher scores in PCS and the affective dimensions of SF-MPQ. Widespread pain, catastrophizing, and negative emotions, which influence descending inhibitory pain pathways in chronic pain (29, 30), may form a vicious cycle.

When patients present with unilateral complicated pain, the diagnosis of either PIDAP or TN will be more difficult. Because NVC has been considered a main cause of TN, determining whether the presence or absence of NVC might aid in differentiating TN from PIDAP. In the present study, NVC was observed in both patients with TN and PIDAP without a significant between-group difference, as well as in other clinical features. However, detailed conditions of NVC, including compression or displacement of trigeminal nerves, may help differentiate them.

The further analysis of survey factors relating to diagnosis revealed that the pain distribution radiating to the face would be helpful to differentiate between TN and PIDAP. In this study, significantly more patients with TN reported facial pain compared to patients with PIDAP, as previously reported that TN involves pain in both the face and the tooth (11–13). While 69.2% of TN patients reported tooth pain in the present study, with a higher prevalence than that in previous reports (31), patients with PIDAP also experienced facial pain with a higher frequency compared to the previous report (15). Co-occurring pain in both the face and tooth makes diagnosis challenging in this patient population; however, detailed pain distribution may aid in diagnosis.

Moreover, the higher scores of CSI and SSS-8 with a headache history would be the keys to diagnosis, rather than psychiatric comorbidity. Although 38.2% of patients with PIDAP showed psychiatric comorbidities in this study, most of them had depressive disorders and rarely schizophrenia or bipolar disorder, consistent with previous reports (15, 28). Psychiatric comorbidities were more commonly observed in PIDAP than in TN; however, no significant difference in the scores of SDS and PCS was observed in this study. On the contrary, patients with PIDAP presented significantly higher scores of CSI and SSS-8, as well as a higher frequency of a headache history, which aligns with previous studies (14, 15, 28). The detailed analysis of CSI and SSS-8 detected that patients with PIDAP scored significantly higher on items related to fatigue and a higher tendency related to sleep disturbance compared to patients with TN. The bidirectional link between sleep and pain has been reported to enhance the importance of addressing sleep quality in pain management (32). Central sensitization may be involved in perpetuating such chronic pain cycles (33), as well as impaired descending pain inhibition (29). The CSI reflects the degree of this sensitization (21), and SSS-8 also relates to central sensitization, assessing the severity of somatic symptoms (23). The vicious cycle between physical burden, sleep disturbance, and chronic pain might be formed, involving the central nervous system, in patients with PIDAP. Therefore, the differences in CSI and SSS-8 between PIDAP and TN may reflect a pathophysiological mechanism rather than simply a result of psychiatric comorbidities. In addition, the previous study suggests the importance of assessing central sensitization besides conventional pain questionnaires (34). Assessing it using CSI and SSS-8 would be useful for distinguishing PIDAP from TN, consequently, for pain management in patients with PIDAP.

Furthermore, detailed pain characteristics, “shooting” and “stabbing” pain, may differentiate TN from PIDAP. The typical characteristic pain descriptors are generally “electric-like shock” or “stab-like” for TN (11), and “heavy” or “throbbing” for PIDAP (4, 6). To compare each item of SF-MPQ, depending on the final diagnosis, revealed that while patients with TN more frequently reported “shooting” and “stabbing” pain, “tender” or “aching” descriptors were severely complained in both patient groups. Additionally, some patients with TN presented with “heavy” pain and “throbbing”, which was rather more frequent than in patients with PIDAP, while some patients with PIDAP presented with “shooting” pain. Although their typical pain characteristics were controversially shown, suggesting substantial complexity, assessing the severity of each pain expression in SF-MPQ may be useful for diagnosis.

On the point of treatment response, Amitriptyline was effective in 67.5% of PIDAP patients, while carbamazepine was effective in 78.5% of TN patients. The 42.9% patients with TN who were prescribed amitriptyline also showed improvement. These results indicate that the first-line medications typically used in each disorder were effective even though their pain symptoms were complicated by some similarities. Amitriptyline enhances descending inhibitory pathways by blocking serotonin and norepinephrine reuptake in the synapses (35, 36). The exact mechanism of carbamazepine remains partially unclear, but it is believed to inhibit voltage-gated sodium channels in excitatory neurons, thereby suppressing the conduction of action potentials (8, 35, 37). Recently, the involvement of central mechanisms with changes of sodium channels, leading to heightened neuronal excitability and ectopic firing of trigeminal nerve fibers (38), has been considered. In addition to strengthening the descending inhibitory pathway, amitriptyline may have ameliorated persistent pain in TN by inhibiting voltage-dependent sodium channels (39, 40), thereby suppressing voltage propagation. Further investigations are needed to clarify central and peripheral interactions producing complex pain symptoms in both PIDAP and TN.

In this study, the effective dose range of amitriptyline for PIDAP was 5–60 mg. However, there was no correlation between the dose required for improvement and the initial VAS. A previous study also reported no correlation between amitriptyline blood levels and the degree of symptom improvement in some types of chronic pain (41). More research is needed to clarify the pharmacological mechanisms and factors associated with the optimal dose for treating patients with PIDAP.

This study has several limitations. First, the sample size of patients with TN was small, and many were referred to our department specifically because idiopathic sensations were suspected. This may have introduced a bias, particularly compared to classic trigeminal neuralgia. Nonetheless, our department's specialty allowed us to investigate complicated pain presentations, suggesting the need for larger, more broadly representative samples in future research. Second, due to the skewness of sample size, the analysis for interactions between NVC and other clinical characteristics was only conducted depending on the presence or absence of NVC, but not on the degree of trigeminal nerve compression by the blood vessels. The previous research has indicated that the compression between the trigeminal nerve and the offending blood vessel is significantly more severe on the symptomatic side in TN (14). Third, the measure of medication efficacy was limited to improvement rates, effective dose range, and the time to improvement. Further investigations are needed to clarify the underlying pharmacological mechanisms and factors relating to prognosis and optimal dosing.

## 5 Conclusion

In this study, patients with bilateral persistent pain in their teeth, dentoalveolar and/or face had significantly higher scores in PCS and the affective dimensions of SF-MPQ compared to patients with unilateral persistent pain. This may indicate the interaction between widespread pain symptoms and pain catastrophizing. There was no significant clinical difference regardless of the presence of NVC. However, patients with TN experienced significantly stronger “shooting” and “stabbing” pain with more severe compressed or displaced trigeminal nerves, while patients with PIDAP showed significantly higher CSI and SSS-8 scores. These results suggest that relying solely on examination for NVC is insufficient to diagnose PIDAP or TN in patients with complicated persistent pain in their teeth, dentoalveolar, or facial region. Instead, careful assessment of pain quality using SF-MPQ, along with evaluation of central sensitization and somatic symptoms in CSI and SSS-8, as well as detailed neurovascular examinations, may be crucial for differentiating in this patient population.

## Data Availability

The original contributions presented in the study are included in the article, further inquiries can be directed to the corresponding author.

## References

[1] Okeson JP. Nonodontogenic toothache. Tex Dent J. (2000) 117:64–74. Available online at: http://www.ncbi.nlm.nih.gov/pubmed/11858065 (Accessed July 5, 2025). 11858065

[2] Okeson JP Falace DA. Nonodontogenic toothache. Dent Clin North Am. (1997) 41:367–83. 10.1016/S0011-8532(22)00091-X 9142490

[3] Coulter J Nixdorf DR. A review of persistent idiopathic dentoalveolar pain (formerly PDAP/atypical odontalgia). Oral Surg. (2020) 13:371–8. 10.1111/ORS.12472

[4] Benoliel R May A Svensson P Pigg M Law A Nixdorf D International classification of orofacial pain, 1st edition (ICOP). Cephalalgia. (2020) 40:129–221. 10.1177/0333102419893823 32103673

[5] Takenoshita M Miura A Shinohara Y Mikuzuki R Sugawara S Tu TTH Clinical features of atypical odontalgia; three cases and literature reviews. Biopsychosoc Med. (2017) 11:21. 10.1186/s13030-017-0106-8 28785306 PMC5541751

[6] Toyofuku A. Psychosomatic problems in dentistry. Biopsychosoc Med. (2016) 10:14. 10.1186/s13030-016-0068-2 27134647 PMC4851772

[7] Headache Classification Committee of the International Headache Society (IHS) The International Classification of Headache Disorders, 3rd edition. Cephalalgia. (2018) 38:1–211. 10.1177/0333102417738202 29368949

[8] Araya EI Claudino RF Piovesan EJ Chichorro JG. Trigeminal neuralgia: basic and clinical aspects. Curr Neuropharmacol. (2020) 18:109–19. 10.2174/1570159X17666191010094350 31608834 PMC7324879

[9] Maarbjerg S Wolfram F Heinskou TB Rochat P Gozalov A Brennum J Persistent idiopathic facial pain—a prospective systematic study of clinical characteristics and neuroanatomical findings at 3.0 tesla MRI. Cephalalgia. (2017) 37:1231–40. 10.1177/0333102416675618 27789649

[10] Bendtsen L Zakrzewska JM Heinskou TB Hodaie M Leal PRL Nurmikko T Advances in diagnosis, classification, pathophysiology, and management of trigeminal neuralgia. Lancet Neurol. (2020) 19:784–96. 10.1016/S1474-4422(20)30233-7 32822636

[11] Maarbjerg S Gozalov A Olesen J Bendtsen L. Concomitant persistent pain in classical trigeminal neuralgia—evidence for different subtypes. Headache. (2014) 54:1173–83. 10.1111/head.12384 24842632

[12] Spencer CJ Neubert JK Gremillion H Zakrzewska JM Ohrbach R. Toothache or trigeminal neuralgia: treatment dilemmas. J Pain. (2008) 9:767–70. 10.1016/j.jpain.2008.07.001 18722958

[13] Zakrzewska JM Wu J Mon-Williams M Phillips N Pavitt SH. Evaluating the impact of trigeminal neuralgia. Pain. (2017) 158:1166–74. 10.1097/J.PAIN.0000000000000853 28114183

[14] Suzuki M Yoshino N Shimada M Tetsumura A Matsumura T Fukayama H Trigeminal neuralgia: differences in magnetic resonance imaging characteristics of neurovascular compression between symptomatic and asymptomatic nerves. Oral Surg Oral Med Oral Pathol Oral Radiol. (2015) 119:113–8. 10.1016/J.OOOO.2014.09.013 25442253

[15] Kawasaki K Sugawara S Watanabe K Hong C Tu TTH Watanabe T Differences in the clinical characteristics of persistent idiopathic facial pain (atypical odontalgia) patients with or without neurovascular compression of the trigeminal nerve. Pain Med. (2020) 21:814–21. 10.1093/pm/pnz300 32040150 PMC7139210

[16] Campbell FG Graham JG Zilkha KJ. Clinical trial of carbazepine (tegretol) in trigeminal neuralgia. J Neurol Neurosurg Psychiatry. (1966) 29:265–7. 10.1136/JNNP.29.3.265 5327969 PMC496031

[17] Tu TTH Miura A Shinohara Y Mikuzuki L Kawasaki K Sugawara S Pharmacotherapeutic outcomes in atypical odontalgia: determinants of pain relief. J Pain Res. (2019) 12:831–9. 10.2147/JPR.S188362 30881094 PMC6398971

[18] American Psychiatric Association. Diagnostic and Statistical Manual of Mental Disorders. Washington, DC: American Psychiatric Association Publishing (2013). 10.1176/APPI.BOOKS.9780890425596

[19] Melzack R Terrence C Fromm G Amsel R. Trigeminal neuralgia and atypical facial pain: use of the McGill pain questionnaire for discrimination and diagnosis. Pain. (1986) 27:297–302. 10.1016/0304-3959(86)90157-0 3808740

[20] Melzack R. The short-form McGill pain questionnaire. Pain. (1987) 30:191–7. 10.1016/0304-3959(87)91074-8 3670870

[21] Mayer TG Neblett R Cohen H Howard KJ Choi YH Williams MJ The development and psychometric validation of the central sensitization inventory. Pain Pract. (2012) 12:276–85. 10.1111/J.1533-2500.2011.00493.X 21951710 PMC3248986

[22] Gierk B Kohlmann S Kroenke K Spangenberg L Zenger M Braḧler E The somatic symptom scale-8 (SSS-8): a brief measure of somatic symptom burden. JAMA Intern Med. (2014) 174:399–407. 10.1001/jamainternmed.2013.12179 24276929

[23] Hashimoto K Takeuchi T Hiiragi M Koyama A Nakamura Y Hashizume M. Utility and optimal cut-off point of the somatic symptom scale-8 for central sensitization syndrome among outpatients with somatic symptoms and related disorders. Biopsychosoc Med. (2022) 16:24. 10.1186/S13030-022-00253-2 36434700 PMC9694559

[24] Zung WW. A self-rating depression scale. Arch Gen Psychiatry. (1965) 12:63–70. 10.1001/archpsyc.1965.01720310065008 14221692

[25] Sullivan MJL Bishop SR Pivik J. The pain catastrophizing scale: development and validation. Psychol Assess. (1995) 7:524–32. 10.1037/1040-3590.7.4.524

[26] Watanabe K Watanabe M Takao C Hong C Liu Z Suga T Clinical characteristics of predominantly unilateral oral cenesthopathy with and without neurovascular contact. Front Neurol. (2021) 12:744561. 10.3389/fneur.2021.744561 34616358 PMC8488299

[27] Guy W. ECDEU Assessment Manual for Psychopharmacology. Rockvill, MD: US Department of Health, Education and Welfare Public Health Service Alcohol, Drug Abuse, and Mental Health Administration (1976).

[28] Miura A Tu TTH Shinohara Y Mikuzuki L Kawasaki K Sugawara S Psychiatric comorbidities in patients with atypical odontalgia. J Psychosom Res. (2018) 104:35–40. 10.1016/J.JPSYCHORES.2017.11.001 29275783

[29] Bushnell MC Čeko M Low LA. Cognitive and emotional control of pain and its disruption in chronic pain. Nat Rev Neurosci. (2013) 14:502–11. 10.1038/NRN3516 23719569 PMC4465351

[30] Miki T Nishigami T Takebayashi T Yamauchi T. Association between central sensitivity syndrome and psychological factors in people with presurgical low back pain: a cross-sectional study. J Orthop Sci. (2021) 26:337–42. 10.1016/j.jos.2020.03.017 32331990

[31] Tripathi M Sadashiva N Gupta A Jani P Pulickal SJ Deora H Please spare my teeth! dental procedures and trigeminal neuralgia. Surg Neurol Int. (2021) 11:455. 10.25259/SNI_729_2020 PMC7771490 33408940

[32] Seiger AN Penzel T Fietze I. Chronic pain management and sleep disorders. Cell Rep Med. (2024) 5:101761. 10.1016/j.xcrm.2024.101761 39413729 PMC11513819

[33] Campbell CM Buenaver LF Finan P Bounds SC Redding M McCauley L Sleep, pain catastrophizing, and central sensitization in knee osteoarthritis patients with and without insomnia. Arthritis Care Res (Hoboken). (2015) 67:1387–96. 10.1002/ACR.22609 26041510 PMC4580506

[34] Liu Y Chuinsiri N Lefaucheur J-P. The intrinsic reason why LANSS, DN4, and PainDETECT questionnaires cannot distinguish neuropathic pain from nociplastic pain. Front Pain Res. (2025) 6:1658126. 10.3389/FPAIN.2025.1658126 PMC12408643 40919440

[35] Marcianò G Vocca C Evangelista M Palleria C Muraca L Galati C The pharmacological treatment of chronic pain: from guidelines to daily clinical practice. Pharmaceutics. (2023) 15:1165. 10.3390/PHARMACEUTICS15041165 37111650 PMC10144480

[36] Sindrup SH Otto M Finnerup NB Jensen TS. Antidepressants in the treatment of neuropathic pain. Basic Clin Pharmacol Toxicol. (2005) 96:399–409. 10.1111/J.1742-7843.2005.PTO_96696601.X 15910402

[37] Di Stefano G La Cesa S Truini A Cruccu G. Natural history and outcome of 200 outpatients with classical trigeminal neuralgia treated with carbamazepine or oxcarbazepine in a tertiary centre for neuropathic pain. J Headache Pain. (2014) 15:34. 10.1186/1129-2377-15-34 24912658 PMC4067104

[38] Luiz AP Kopach O Santana-Varela S Wood JN. The role of Nav1.9 channel in the development of neuropathic orofacial pain associated with trigeminal neuralgia. Mol Pain. (2015) 11:72. 10.1186/S12990-015-0076-4 26607325 PMC4658751

[39] Kang IS Cho JH Lee MG Jang IS. Modulation of tetrodotoxin-resistant na+ channels by amitriptyline in dural afferent neurons. Eur J Pharmacol. (2018) 838:69–77. 10.1016/j.ejphar.2018.09.006 30194938

[40] Liang J Liu X Pan M Dai W Dong Z Wang X Blockade of Nav1.8 currents in nociceptive trigeminal neurons contributes to anti-trigeminovascular nociceptive effect of amitriptyline. Neuromolecular Med. (2014) 16:308–21. 10.1007/S12017-013-8282-6 24292897

[41] Rascol O Tran MA Bonnevialle P Belin J Cotonat J Guiraud-Chaumeil B Lack of correlation between plasma levels of amitriptyline (and nortriptyline) and clinical improvement of chronic pain of peripheral neurologic origin. Clin Neuropharmacol. (1987) 10:560–4. 10.1097/00002826-198712000-00008 3427563

